# A Benzimidazole-Based N-Heterocyclic Carbene Derivative Exhibits Potent Antiproliferative and Apoptotic Effects against Colorectal Cancer

**DOI:** 10.3390/medicina60091379

**Published:** 2024-08-23

**Authors:** Sarah Al-Nasser, Maha Hamadien Abdulla, Noura Alhassan, Mansoor-Ali Vaali-Mohammed, Suliman Al-Omar, Naceur Hamdi, Yasser Elnakady, Sabine Matou-Nasri, Lamjed Mansour

**Affiliations:** 1 Zoology Department, College of Science, King Saud University, Riyadh 11451, Saudi Arabia; smn-ss@hotmail.com (S.A.-N.); syalomar@ksu.edu.sa (S.A.-O.); yelnakady@ksu.edu.sa (Y.E.); 2 Colorectal Research Chair, Department of Surgery, College of Medicine, King Saud University, Riyadh 11472, Saudi Arabiamansooralicytogene@gmail.com (M.-A.V.-M.); 3 Research Laboratory of Environmental Sciences and Technologies (LR16ES09), Higher Institute of Environmental Sciences and Technology, University of Carthage, Hammam-Lif 2050, Tunisia; hamdi.naceur@isste.ucar.tn; 4 Blood and Cancer Research Department, King Abdullah International Medical Research Center (KAIMRC), King Saud bin Abdulaziz University for Health Sciences (KSAU-HS), Ministry of National Guard-Health Affairs (MNG-HA), Riyadh 11481, Saudi Arabia; matouepnasrisa@mngha.med.sa; 5 Biosciences Department, Faculty of the School of Systems Biology, George Mason University, Manassas, VA 20110, USA

**Keywords:** antiproliferative, apoptosis, colorectal cancer, 1-isobutyl-benzimidazole chloride, metastasis, migration, combination therapy

## Abstract

*Background and Objectives*: Colorectal cancer (CRC) remains a major global health issue. Although chemotherapy is the first-line treatment, its effectiveness is limited due to drug resistance developed in CRC. To overcome resistance and improve the prognosis of CRC patients, investigating new therapeutic approaches is necessary. *Materials and Methods*: Using human colorectal adenocarcinoma (HT29) and metastatic CRC (SW620) cell lines, the potential anticancer properties of a newly synthesized compound 1-(Isobutyl)-3-(4-methylbenzyl) benzimidazolium chloride (IMBZC) were evaluated by performing MTT cytotoxicity, cell migration, and colony formation assays, as well as by monitoring apoptosis-related protein and gene expression using Western blot and reverse transcription–quantitative polymerase chain reaction technologies. *Results:* Tested at various concentrations, the half-maximal inhibitory concentrations (IC_50_) of IMBZC on HT29 and SW620 cell growth were determined to be 22.13 µM (6.97 μg/mL) and 15.53 µM (4.89 μg/mL), respectively. IMBZC did not alter the cell growth of normal HEK293 cell lines. In addition, IMBZC inhibited cell migration and significantly decreased colony formation, suggesting its promising role in suppressing cancer metastasis. Mechanistic analyses revealed that IMBZC treatment increased the expression of pro-apoptotic proteins p53 and Bax, while decreasing the expression of anti-apoptotic proteins Bcl-2 and Bcl-xL, thus indicating the induction of apoptosis in IMBZC-treated CRC cells, compared to untreated cells. Additionally, the addition of IMBZC to conventional chemotherapeutic drugs (i.e., 5-fluorouracil, irinotecan, and oxaliplatin) resulted in an increase in the cytotoxic potential of the drugs. *Conclusions*: This study suggests that IMBZC has substantial anticancer effects against CRC cells through its ability to induce apoptosis, inhibit cancer cell migration and colony formation, and enhance the cytotoxic effects of conventional chemotherapeutic drugs. These findings indicate that IMBZC could be a promising chemotherapeutic drug for the treatment of CRC. Further research should be conducted using in vivo models to confirm the anti-CRC activities of IMBZC.

## 1. Introduction

Colorectal cancer (CRC) is the third most frequent cancer worldwide and the second leading cause of cancer-related death. It is considered one of the most dangerous and burdensome tumors in the world [1]. In recent years, the global prevalence of colorectal cancer has increased at an alarming rate [2]. Patients diagnosed with CRC accounted for 10% of global cancer incidence and 9.4% of cancer deaths in 2020 and are projected to reach 3.2 million by 2040 [2,3]. Smoking, poor diet, excessive alcohol consumption, physical inactivity, and excess body weight account for more than half of all cases and deaths [4,5]. Unfortunately, CRC often remains asymptomatic until later stages, at which point it becomes aggressive, malignant, and prone to spread [2]. As a result, the majority of patients are diagnosed at an advanced stage when symptoms such as rectal bleeding, anemia, or stomach discomfort manifest. When the tumor is localized at the time of diagnosis, surgery or radiotherapy is the preferred treatment, along with the development of molecularly targeted therapy and immunotherapy (i.e., immune checkpoint inhibitors). However, the systemic approach to chemotherapy remains essential for effective cancer management [5]. The main chemotherapeutic drugs used in CRC patients are platinum-based agents (i.e., oxaliplatin, cisplatin, carboplatin) and fluoropyrimidines (i.e., 5-fluorouracil, capecitabine) [6]. The main fundamental mechanisms of most chemotherapeutic drugs such as oxaliplatin (OXA), 5-fluorouracil (5-FU), and irinotecan (IRI) lead to DNA damage by forming DNA strand breaks, disrupt DNA/RNA synthesis, and inhibit topoisomerase I activity, which subsequently inhibits neoplastic cell proliferation [7,8]. So far, the 5-year survival rate of patients with early-stage, locally advanced, and metastatic CRC is estimated to reach 90%, 70%, and 15%, respectively (Wang et al., 2022). Thus, although chemotherapy represents a mainstay clinical intervention, its effectiveness is hampered by the emergence of drug resistance. For instance, IRI was initially licensed for use as a second-line monotherapy in patients with metastatic CRC who had previously failed 5-FU-based therapy [5]. Therefore, there is an urgent need to search for novel therapeutic agents to circumvent resistance and improve the clinical outcomes of CRC patients.

N-heterocyclic carbenes (NHCs) are nitrogen-containing heterocyclic compounds with one bivalent carbon atom and six valence electrons [9]. Due to their inherent qualities, NHCs have recently attracted much attention because they are readily accessible and their physicochemical properties can be easily fine-tuned by changing the wingtip N-substituents, thereby enabling the production of very stable metal complexes [10]. NHCs are commonly used as powerful and efficient nucleophilic catalysts [11]. Organocatalysis with NHCs is a sophisticated approach to rapidly produce physiologically and medicinally relevant molecules from simple and affordable substrates [12]. Recent studies have highlighted the potential of NHC ligands as carriers of anticancer drugs [13]. The focus on using this class of ligands to construct physiologically relevant carbene complexes stems from the core moiety, benzimidazole, which is more biologically accessible as a component of multiple metalloenzymes and proteins [14]. Benzimidazole derivatives are chemical compounds that have a similar structure to naturally occurring nucleotides. This similarity enables them to possibly interact with large biological molecules like proteins, enzymes, and receptors [15]. In addition, the imidazole ring system is a fundamental structural pattern seen in a wide range of biologically active natural compounds and pharmaceuticals. The imidazole ring system contains two nitrogen atoms, which allows it to engage in hydrogen bonding, dipole–dipole interactions, and metal chelation. These interactions are crucial for molecular recognition and binding to biological targets.

Moreover, the inclusion of imidazole or other azole heterocycles, like oxadiazole, in drug-like compounds has been demonstrated to modulate their physicochemical characteristics, such as polarity, flexibility, and metabolic profile [16]. This modulation can improve the pharmacokinetic and pharmacodynamic properties of the compounds, enhancing their suitability for application in medicinal therapies [17].

The purpose of the current study was to evaluate the potential anti-CRC effects of benzimidazolium salts, a 1-(Isobutyl)-3-(4-methylbenzimidazolium) chloride compound (named IMBZC). This study employed colorectal adenocarcinoma HT29 and metastatic CRC (mCRC) SW620 cell lines as CRC models for this in vitro investigation. To evaluate the effect of IMBZC on these cell lines, several assays were carried out, including single and combined cytotoxicity assays with conventional chemotherapeutic agents (i.e., OXA, 5-FU, IRI), cell migration analysis, a colony formation assay, Western blot analysis, and reverse transcription–quantitative polymerase chain reaction (RT-qPCR). This investigation provides insight into the anti-CRC potential of IMBZC and the associated underlying cellular and molecular mechanisms. The results of this study would establish a solid basis for further investigation and advancement. Successful preclinical in vivo research conducted on animal models could provide evidence to support the progression of IMBZC into clinical trials, where its effectiveness as an innovative treatment for colorectal cancer can be evaluated.

## 2. Materials and Methods

### 2.1. Reagents

All reagents were purchased from Sigma-Aldrich (St. Louis, MO, USA) unless indicated otherwise.

### 2.2. Cell Culture

Human colorectal adenocarcinoma HT29 (#HTB-38), mCRC SW620 (#CCL-277), and normal embryonic kidney HEK293 (#CRL-1573) cell lines were obtained from the American Type Culture Collection (ATCC, Manassas, VA, USA) and grown in complete Dulbecco’s modified Eagle’s medium (DMEM) (#1200252, Gibco^®^, Thermo Fisher Scientific, Waltham, MA, USA) supplemented with 10% fetal bovine serum (FBS, #10270106, Gibco^®^) and 1% antibiotic (penicillin/streptomycin) solution (#10378016, Gibco^®^). The cells were cultured at 37 °C in a 5% CO_2_ incubator maintaining a humidified atmosphere. Every 2–3 days, the cells were passaged at confluence using 0.25% trypsin/EDTA (#25200056, Gibco^®^) and were used between passages 2 and 3 throughout this study.

### 2.3. IMBZC Synthesis and Preparation

**Figure 1:**
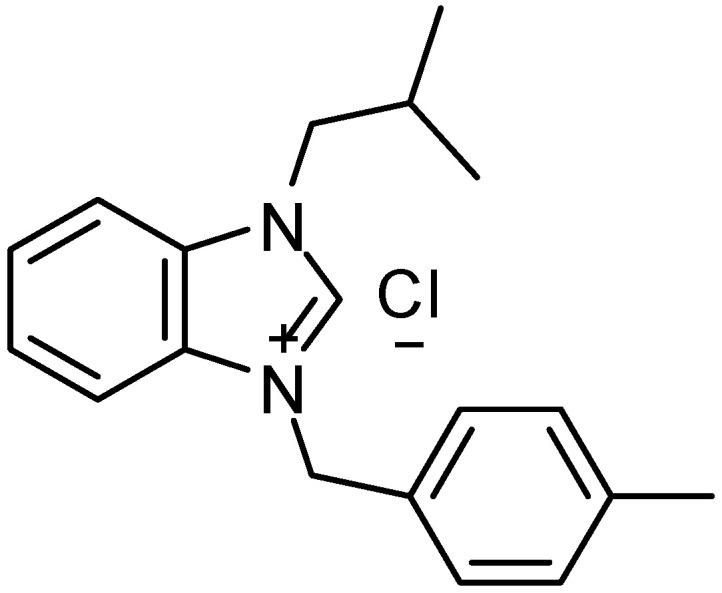
Chemical structure of 1-(Isobutyl)-3-(4-methylbenzyl) benzimidazolium chloride (IMBZC) (MW = 314.85 g/mol).

A 1-(Isobutyl)-3-(4-methylbenzyl) benzimidazolium chloride (IMBZC) (MW = 314.85 g/mol) compound was synthesized and prepared as previously described [18]. Briefly, the synthesis of IMBZC was created by reacting 1 mmol of N-(isobutyl)-benzimidazole with 1.1 mmol of chloro toluene, an alkyl chloride, in 5 mL of dimethylformamide (DMF) at 80 °C for 24 h. Diethyl ether (15 mL) was added to produce a white crystalline solid, which was then filtered off (yield 87%). The solid was washed with diethyl ether (3 × 10 mL) and dried under vacuum, and the crude product was recrystallized from dichloromethane/diethyl ether (1:3 ratio) The IMBZC compound was dissolved in methanol to prepare a 10 mg/mL stock solution and stored at room temperature until use. The structure of IMBZC was previously characterized by elemental analyses, including ^1^H nuclear magnetic resonance (NMR), ^13^C NMR, and infrared (IR) spectroscopy techniques [18] (Figure 1).

### 2.4. MTT Cytotoxicity Assay

Cell lines (i.e., HT29, SW620, and HEK293) were plated at 5 × 10^3^ cells per well in complete medium in 96-well culture plates for 24 h of incubation. Cells were then cultured in the absence (i.e., untreated control cells) or presence of various doses of IMBZC—3.97, 7.94, 15.88, 23.82, and 31.76 µM (equivalent to 1.25, 2.5, 5, 7.5, and 10 µg/mL)—for 24 h of incubation. To assess the cytotoxic effect of conventional CRC chemotherapeutic drugs combined with IMBZC, cells were exposed to 5-FU, IRI, and OXA, tested at 2.5, 5, and 10 µM. In total, 10 µL of MTT (3-(4,5-dimethylthiazol-2-yl)-2,5-diphenyl tetrazolium bromide) solution was added to the cells 2 h before the 24 h completion, which resulted in violet formazan crystal formation in metabolically active cells. After that, 100 μL of isopropanol was added to each well to dissolve the crystals. Colorimetric absorbance was measured at 540 nm using a Synergy^™^ 2 multi-mode microplate reader (BioTek Inc., Winooski, VT, USA). For each condition, experiments were carried out in triplicate as previously described [19]. The value of half-maximal inhibitory concentrations (IC_50_) leading to a 50% decrease in cell viability was also determined. 

### 2.5. Scratch Wound-Healing Assay

HT29 and SW620 cells were seeded at 1 × 10^6^ cells per well in complete medium in 6-well plates and incubated until confluence. A sterile 200 µL pipette tip was used to scratch a wound in the center of each confluent cell monolayer. The old medium was replaced with fresh complete culture medium to remove dislodged cells. Wells containing cells exposed only to complete medium were used as controls (i.e., untreated cells). IBMZC tested at 15.88 µM (5 µg/mL) and 23.82 µM (10 µg/mL) was added to wells containing HT29 cells, while IBMZC tested at 7.94 µM (2.5 µg/mL) and 15.88 µM (5 µg/mL) was added to wells with SW620 cells. The healing area was measured to assess the capacity and motility of the cells to close the wound. This was accomplished by photographing the scratch at point 0 (t0) using an inverted light microscope (MICROS, Sundew MCX 1600, St. Veit an der Glan, Austria). The plates were then incubated at 37 °C for 24 h (t final) in a CO_2_ incubator (Sanyo, MCO175, Marshall Scientific, Hampton, NH, USA). The second photos were taken after the final incubation and the average gap widths at t0 and t final were qualitatively evaluated compared to untreated control cells [13].

### 2.6. Clonogenic Assay

The colony formation experiment was performed as described previously by Al-Khayal [20]. Briefly, HT29 and SW620 cells were cultured at 500 cells per well in 6-well plates with complete medium. After adding IMBZC (15.88 µM and 23.82 µM) to the cells for 24 h, each well was replaced with fresh medium then cultured for 10–12 days in a CO_2_ incubator. Colonies were fixed with 4% paraformaldehyde and stained with 0.05% crystal violet. The colonies consisting of more than 50 cells were quantified using a microscope.

### 2.7. Real-Time Reverse Transcription–Quantitative Polymerase Chain Reaction (RT-qPCR)

HT29 and SW620 cells (1 × 10^6^) were grown in complete medium for 24 h in a 100 mm dish up to 60% confluency. The following day, cells were treated with or without 7.94, 15.88, or 23.82 µM (equivalent to 2.5, 5 and 10 µg/mL) of IMBZC for an additional 24 h incubation. The RNeasy^®^ Mini kit (Qiagen, Hilden, Germany) was used to extract total RNA from untreated and treated cells. The FIREScript^®^ RT cDNA synthesis kit containing Mix with oligo (dT) and a Random Primers kit (Solis BioDyne, Tartu, Estonia) were used to synthesize the complementary (c)DNA. The ratio of absorbance readings of 260/280 (1.8–2.0) was calculated to evaluate RNA quality using a NanoDrop^®^ ND-2000 UV-VIS spectrophotometer (Thermo Fisher Scientific, Inc.). SYBR^™^ Green PCR Master Mix (#4385612, Thermo Fisher Scientific) was used for qPCR analysis using the Applied Biosystems^™^ ViiA^™^ 7 real-time PCR system (Thermo Fisher Scientific, Inc.). *B-cell lymphoma (BCL)-2*, *BCL-extra-large (BCL-xL)*, *BCL-associated X protein (BAX)*, and *tumor suppressor TP53* mRNA expression levels were adjusted to *glyceraldehyde-3-phosphate dehydrogenase* (*GAPDH)* used as an internal control for real-time RT-qPCR. The sequences of primer pairs are shown in Table 1. A negative control lacking a cDNA template was used for each gene analysis. All reactions were carried out in triplicate as described previously [20].

### 2.8. Western Blot Technology

HT29 and SW620 cells were cultured in complete medium for 24 h before being in the presence or absence of 7.94, 15.88, and 31.76 µM (2.5, 5, and 10 μg/mL) of IMBZC. Cells were collected the next day and washed twice with phosphate-buffered saline (PBS). Radio-immunoprecipitation assay (RIPA) lysis buffer was used to generate total cell lysates from cell pellets for 15 min at 4 °C. After that, the entire mixture was centrifuged at 15,000 g; then, the supernatant containing the soluble proteins was collected and the protein concentration was determined using Bradford reagent on a SmartSpec^™^ Plus spectrophotometer (Bio-Rad Laboratories, Hercules, CA, USA). For sodium dodecyl sulfate–polyacrylamide gel electrophoresis (SDS-PAGE), 10–20 μL of proteins was loaded (4–20% Mini-Protean TGX precast gels, Bio-Rad Laboratories). Using the rapid protein transfer system (Bio-Rad Laboratories), protein separated on precast gels was transferred to a polyvinylidene fluoride (PVDF) membrane. Following this, the membranes containing the transferred proteins were saturated with blocking buffer for 1 h at room temperature. Membranes were then washed twice in PBS with 0.1% Tween-20 (PBST). Membranes were then incubated with primary antibodies directed against Bcl-2 (#A86278, Antibodies.com, Cambridge, UK), Bcl-xL (#A36576, Antibodies.com), Bax (#A249822, Antibodies.com), TP53 (#A250176, Antibodies.com), and β-Actin (#sc-69879, Santa Cruz Biotechnology, Dallas, TX, USA) with 1:1000 dilution in blocking buffer. After overnight incubation with the primary antibodies on an orbital shaker at 4 °C, the membranes were washed twice with PBST before being incubated for 1 h at room temperature with rabbit anti-mouse IgG-horseradish peroxidase (HRP) secondary antibodies (#A301453, Antibodies.com) (1:10,000 dilution). The chemiluminescence signal was detected by a high-sensitivity enhanced chemiluminescence (ECL) substrate kit (Millipore, Thermo Fisher Scientific), according to the manufacturer’s instructions. The C-DiGit^®^ blot scanner (LI-COR Biosciences, Lincoln, NE, USA) was used to detect the signals [21].

### 2.9. Statistical Analysis

All experiments were performed in triplicate and data were provided as the mean ± standard deviation (SD) of three independent experiments. Student’s *t*-test was used to assess differences between the control and treated groups. A *p*-value of less than 0.05 was used to determine statistical significance.

## 3. Results

### 3.1. IMBZC Compound Inhibits CRC Cell Viability in a Dose-Dependent Manner

**Figure 2:**
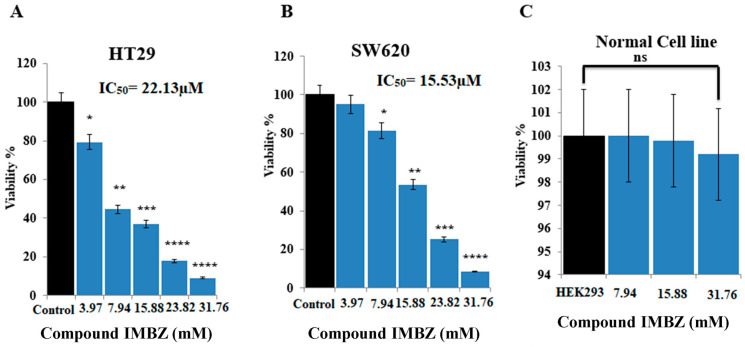
Assessment of the cytotoxic potential of compound IMBZC on colon adenocarcinoma HT29 and mCRC SW620 cells, in comparison with normal HEK293 cells, using MTT assay. Determination of percent viability of (**A**) HT29, (**B**) SW620, and (**C**) HEK923 cells after 24 h of incubation in the absence (i.e., control) or presence of increasing concentrations of (3.97 to 31.76 µM) IMBZC. * *p* < 0.05, ** *p* < 0.01, *** *p* < 0.001, **** *p* < 0.0001 vs. control. (ns: not significant).

The MTT assay was used to determine the cytotoxic impact of the compound IMBZC on CRC cell viability, the main cellular event contributing to tumor development and cancer cell survival potential. Human colon adenocarcinoma HT29 and CRC SW620 cells were exposed to increasing concentrations from 3.97 to 23.82 µM (i.e., 1.25 µg/mL to 10 µg/mL) of IMBZC for 24 h. Compared to the high viability of untreated cells (i.e., control) corresponding to 100%, IMBZC tested at the lowest concentration (3.97 µM) decreased the viability of HT29 cells significantly by 20% (*p* < 0.05), while a decrease of 60% and 90% (*p* < 0.01 and *p* < 0.001) was observed when HT29 cells were exposed to 7.94 to 15.88 µM and 23.82 to 31.76 µM (i.e., 2.5–5 µg/mL and 7.5–10 µg/mL) of IMBZC, respectively (Figure 2A). Regarding mCRC SW620 cells, IMBZC tested at 7.94 µM significantly decreased cell viability by 20% (*p* < 0.05), followed by 50%, 80%, and 90% decreases when SW620 cells were exposed to 15.88, 23.82, and 31.76 µM (5 µg/mL, 7.5 µg/mL, and 10 µg/mL) of IMBZC, respectively (Figure 2B). The IMBZC IC_50_ value for HT29 cells was calculated to be 22.13 µM (6.97 µg/mL), while the IMBZC IC_50_ value for SW620 was 15.53 µM (4.89 µg/mL). Thus, the compound exhibited significant cytotoxicity toward CRC cells in a dose-dependent manner, whereas the percentage viability of normal HEK293 cells was not affected at all IBMZC concentrations tested (Figure 2C).

### 3.2. IMBZC Compound Decreases CRC Cell Migration and Inhibits Colony Formation

**Figure 3:**
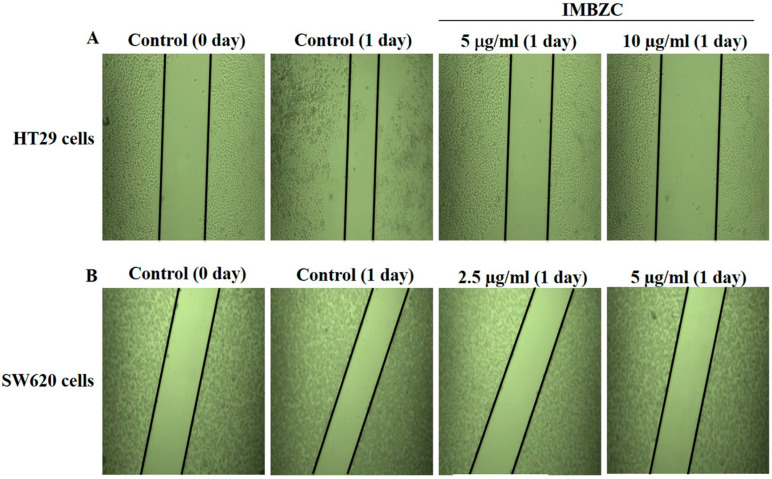
Evaluation of the antiproliferative potential of IMBZC on CRC cell migration using wound healing assay. (**A**) HT29 and (**B**) SW620 cells were seeded in 6-well plates and incubated with complete medium until confluence, and then the cell monolayer was scratched with a sterile 200 µL tip and washed with PBS. The medium was replaced with or without IMBZC then incubated for 24 h. Microscopy was used to examine the cells, and digital images were taken.

The wound healing assay was assessed by monitoring the shrinkage and closure of damaged wounded regions generated by scratches on a cell monolayer. The antiproliferative properties of IMBZC on cancer cell motility were assessed at concentrations close to its IC_50_ values determined for each CRC cell line. As shown in the representative photomicrographs, after 24 h of incubation, the gap size in the wounded HT29 (Figure 3A) cell monolayer treated with IMBZC was nearly one-fold larger than in untreated controls, demonstrating that the IMBZC compound considerably inhibited the migration of both CRC cell lines (Figure 3). In addition, the gap distances in IMBZC-treated HT29 (Figure 3A) cells were progressively larger with increasing concentrations (23.82 and 31.76 µM (5 and 10 µg/mL)) of IMBZC, indicating an inhibitory effect of the compound in a dose-dependent manner (Figure 3). For the SW620 cell lines, the gap size after 1 day of IMBZC treatment is similar to the control on day 0 and slightly larger than the control after 1 day (Figure 3B).

**Figure 4:**
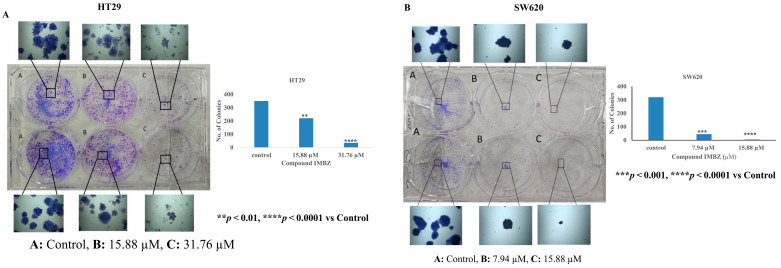
The compound IMBZC inhibits HT29 and SW620 cell-based colony formation. Both HT29 (**A**) and SW620 (**B**) cells were incubated for 10–12 days at 37 °C for colony formation, along with untreated HT29 (**A**.**A**) and SW620 (**B**.**A**) cells (i.e., control). The number of colonies is represented by a bar graph and the data are presented as mean ± SD (N = 3). ** *p* < 0.01, *** *p* < 0.001, and **** *p* < 0.0001 vs. control.

The antiproliferative effect of the IMBZC compound on HT29 and SW620 cells was investigated using a colony formation assay at a similar range of doses (15.88 and 31.76 µM) on HT29 and (7.94 and 15.88 µM) SW620 cells. Compared to untreated colony-generating cells based on CRC cells, IMBZC significantly inhibited colony formation using HT29 (Figure 4A) and SW620 (Figure 4B) cells. Compared to the number of colonies generated in the control, IMBZC significantly decreased HT29 cell-based colony formation by 40% (*p* < 0.01) and 90% (*p* < 0.0001) when tested at 15.88 and 31.76 µM, respectively (Figure 4A). Regarding the SW620 cell line, IMBZC significantly decreased colony formation by 80% (*p* < 0.001) and 98% (*p* < 0.0001) when tested at 7.94 and 15.88 µM, respectively, compared to untreated control cells (Figure 4B).

### 3.3. IMBZC Downregulates the Expression Levels of Anti-Apoptotic Bcl-2 and Bcl-xL While Upregulating Pro-Apoptotic Bax and p53 in a Dose-Dependent Manner

**Figure 5:**
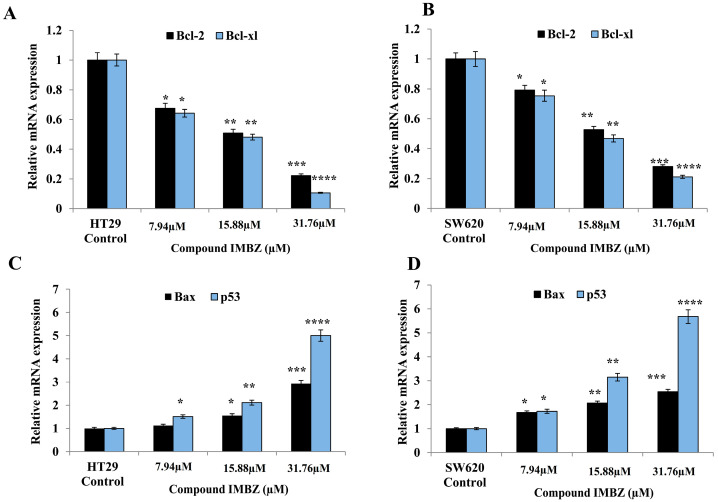
Expression of mRNA levels of anti-apoptotic *BCL-2* and *BCL-xL* (**A**,**B**) and pro-apoptotic *BAX* and *TP53* (**C**,**D**) genes monitored in colorectal adenocarcinoma HT29 (**A**,**C**) and mCRC SW620 (**B**,**D**) (cell lines using RT-qPCR. Bar graphs show the relative gene expression levels calculated as a ratio to *GAPDH*, the internal control, and data are presented as mean ± SD (N = 3) * *p* < 0.05, ** *p* < 0.01, *** *p* < 0.001, **** *p* < 0.0001 vs. control.

**Figure 6:**
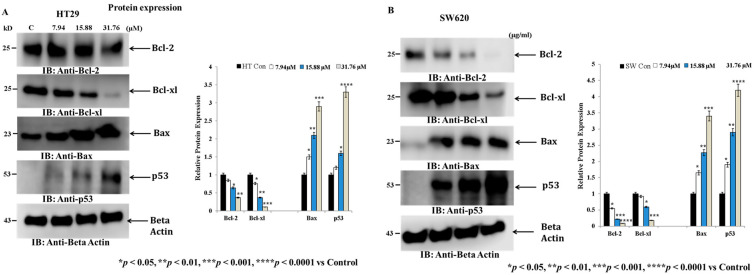
The effect of IMBZC on the anti-apoptotic Bcl-2 and Bcl-xl and pro-apoptotic Bax and p53 protein expression levels. (**A**) HT29 and (**B**) SW620 cells were treated for 24 h at different concentrations (7.94, 15.88 and 31.76 µM) of IMBZC. Anti-Bcl-2, Bcl-xl, p53, and Bax antibodies were used to target these proteins in whole cell lysates. The strength of protein bands was semi-quantified relative to β-actin, used as a loading control, and was presented as relative to protein expression, compared to the untreated control. The bar graphs show the mean ± SD of three independent experiments. * *p* < 0.05, ** *p* < 0.01, *** *p* < 0.001, and **** *p* < 0.0001 vs. control.

Figure 5 shows the expression levels of major anti-apoptotic (i.e., *BCL-2* and *BCL-xL*) and pro-apoptotic (i.e., *TP53*, *BAX*) genes in the CRC cell lines HT29 and SW620 after treatment with various concentrations (7.94, 15.88, and 31.76 µM) of the IMBZC compound, compared to untreated cells (i.e., control). IMBZC significantly decreased *BCL-2* and *BCL-xL* gene expression levels by 30% (*p* < 0.05, Figure 5A) in HT29 cells and by 20% (*p* < 0.05, Figure 5B) in SW620 cells when tested at 7.94 µM, compared to basal levels measured in untreated cells. At high concentrations (15.88 and 31.76 µM), IMBZC significantly inhibited *BCL-2* and *BCL-xL* gene expression levels by 50% (*p* < 0.01) and 80% (*p* < 0.001) in both cell lines, compared to the control (Figure 5). Regarding the pro-apoptotic *BAX* and *TP53* expression levels, IMBZC tested at 7.94 µM significantly upregulated *TP53* expression by 1.5-fold (*p* < 0.05, Figure 4C) in HT29 cells and upregulated *BAX* and *TP53* expression by approximately 1.5-fold (*p* < 0.05, Figure 5D) in SW620 cells, compared to the basal expression levels measured in the control (Figure 5). When tested at 15.88 and 31.76 µM, IMBZC significantly increased pro-apoptotic BAX and TP53 gene expression levels by 1.5–2.0-fold (*p* < 0.05) and 3.0–5.0-fold (*p* < 0.001) in HT29 cells (Figure 5C), while a 2.0–3.0-fold (*p* < 0.01) and 3.0–5.0-fold (*p* < 0.001) increase in these pro-apoptotic gene expression levels was observed in SW620 cells (Figure 5D), compared to the control (Figure 5). Altogether, IBMZC had a stimulatory effect on the pro-apoptotic BAX and TP53 gene expression levels and an inhibitory effect on the anti-apoptotic BCL-2 and BCL-xL gene expression levels in both CRC cells, in a dose-dependent manner (Figure 5). This modulatory effect was confirmed at protein expression levels using Western blot analysis in both HT29 (Figure 6A) and SW620 (Figure 6B) cells.

### 3.4. IMBZC Enhances the Cytotoxic Effects of Conventional CRC Drugs 5-FU, IRI, and OXA

**Figure 7:**
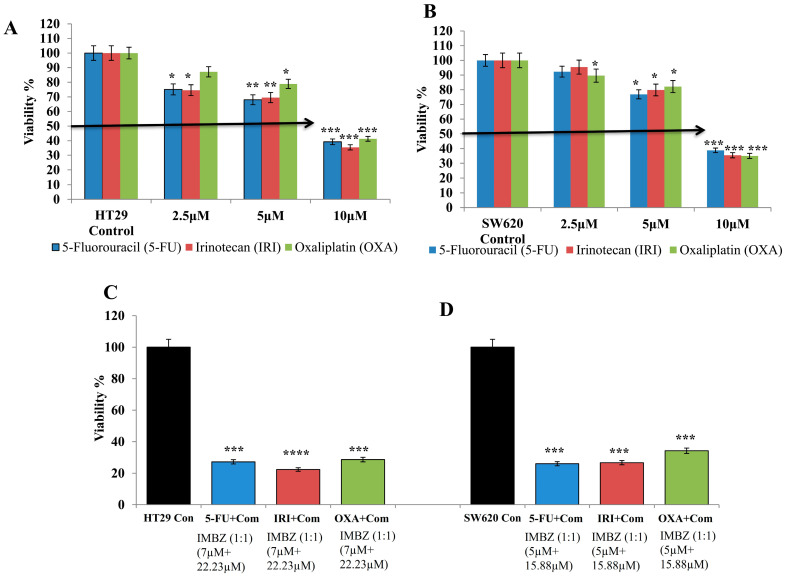
IMBZC potentiates the cytotoxicity of CRC conventional drugs (i.e., 5-FU, IRI and OXA) in (**A**) HT29 and (**B**) SW620 cells. 5-FU, IRI, and OXA drugs were tested at different concentrations (2.5, 5 and 10 µM) for 24 h of incubation. Combinations of conventional drugs with the IMBZC compound on HT29 (**C**) and SW620 (**D**) cell lines. The cell viability percentage was determined using MTT assay. The bar graphs show the mean ± SD of three independent experiments. * *p* < 0.05, ** *p* < 0.01, *** *p* < 0.001, and **** *p* < 0.0001 vs. control.

Determined by an MTT assay, the cytotoxic effect of conventional CRC drugs (i.e., 5-FU, IRI, OXA) on the HT29 cell line is shown in Figure 7A. 5-FU and IRI exhibited a significant cytotoxic effect at all concentrations tested (i.e., 2.5, 5 and 10 µM), while OXA significantly inhibited HT29 cell viability when tested at 5 µM and 10 µM, compared to the control (Figure 7A). Figure 7B shows the cytotoxic effect of these conventional drugs on the SW620 cell line. A significant cytotoxic effect of 5-FU and IRI in SW620 cells was observed when tested at 5 µM and 10 µM, while OXA exhibited a cytotoxic effect at all concentrations tested, compared to the control (Figure 7B). Overall, at the highest concentration (i.e., 10 µM), each chemotherapeutic drug significantly inhibited CRC cell viability by 65–70% (*p* < 0.001) (Figure 7A,B). Combinations of each conventional drug with the IMBZC compound significantly decreased CRC cell viability by 75–80% (Figure 7C), revealing an enhancement of the cytotoxic effect of all the drugs in both cell lines by the addition of IMBZC, compared to the single treatment (Figure 7A,B). For HT29 cells, the most significant effect was obtained with IRI + IMBZC at concentrations of 7 µM + 23.82 µM, respectively, while for SW620 cells, the most significant cytotoxicity was obtained with 5-FU + IMBZC at the concentrations of 5 µM + 15.88 µM, compared to the control (Figure 7C,D).

## 4. Discussion

According to GLOBOCAN 2020, colorectal cancer remains the top cause of cancer-related deaths among men and women, and its incidence and mortality rates are increasing each year [3]. Chemotherapy is the most widely utilized therapeutic strategy against tumors; however, due to its toxicity to normal cells and the progressive increase in cancer cell resistance, there is an urgent need to identify novel drugs as alternative cancer treatments [21,22].

NHC ligands can be easily changed and are efficient σ-donors with a weak π-acceptor [12,23]. NHC ligands might potentially stabilize metal complexes against demetallation under physiological conditions [24]. Several biological studies have found that metal complexes containing NHCs are promising antineoplastic drugs [25,26].

In this study, we investigated the cytotoxicity mechanism of compound IMBZC on CRC cell lines HT29 and SW620 and found that IMBZC decreased the cell viability of human CRC cell lines in a dose-dependent manner. IMBZC was found to exhibit a strong cytotoxic effect on colon adenocarcinoma CRC SW620 cells compared to mCRC SW620 cells, with IC_50_ values of 15.53 µM and 22.13 µM, respectively. However, no cytotoxic effect of IMBZC on the normal cell line (HEK293) was observed, indicating the compound IMBZC as a safe antiproliferative drug that kills highly proliferative cancer cells without damaging normal cells. A study conducted by Habib [26] shows that the colon cell line HCT 116 is more sensitive to NHC salts and less sensitive to Ag(I)-NHC complexes.

The current study even investigated whether IMBZC compound treatment could reduce cancer cell migration in HT-29 and SW620 cells. Our findings revealed that IMBZC significantly inhibited the cell migration of CRC cell lines in a dose-dependent manner. This shows that the IMBZC compound has a potent anti-migration ability and therefore could inhibit cancer metastasis in CRC patients.

The final phase in the tumorigenicity cascade is the colonization of tumor cells in distant tissues [27,28]. IMBZC treatment inhibited cancer cell colony formation, indicating that the compound has potential antimetastatic capabilities against human colorectal adenocarcinoma and mCRC. Interestingly, the colon metastatic cell line SW620 is more sensitive to the chemical IMBZC, resulting in a drastic reduction in the colony formation process than the adenocarcinoma cell line HT29. Further in vitro molecular investigations are needed to pinpoint the altered functions and expression of key proteins underlying IMBZC-inhibited SW620 cell-based colony formation, which would enhance our knowledge for better development of a targeted molecular strategy.

The cell fate toward cell death is determined by the balance of pro-apoptotic (e.g., p53, Bax) and anti-apoptotic proteins (e.g., Bcl-2, Bcl-xl). At the gene expression levels, upregulation of *BAX* and tumor suppressor *TP53* is required for induction of apoptosis in colon cancer cells and, in contrast, overexpression of BCL-2 and BCL-xL is necessary for the suppression of apoptosis [29,30]. In this study, we also confirmed at the protein level that IMBZC increases the expression of pro-apoptotic proteins p53 and Bax, while inhibiting the expression of Bcl-2 and Bcl-xl in human colonic adenocarcinoma and mCRC cells.

The combination studies of standard chemotherapeutic drugs IRI, 5-FU, and OXA with IMBZC increased chemotherapeutic drug efficacy in killing CRC cells, suggesting that IMBZC is a promising and safe anticancer chemotherapeutic agent for CRC patients. Due to the high degree of cancer clonal heterogeneity and intratumoral genetic variability, current treatments have limited anticancer efficacy. As a result, the use of medication combinations is becoming more widespread in the search for better treatments [8,31]. According to an in vitro study conducted by Al-Obeed and colleagues, the combination of medicines increased the effect of doxorubicin on mCRC cell line SW620 [28].

## 5. Conclusions

In conclusion, this study showed that the NHC compound IMBZC effectively inhibits viability through the induction of apoptosis, movement, and colony-forming ability of CRC cell lines. The process involves increasing the expression of pro-apoptotic proteins such as p53 and Bax, while decreasing the expression of anti-apoptotic factors such as Bcl-2 and Bcl-xl. IMBZC was demonstrated to enhance the effects of primary chemotherapeutic drugs against CRC. Further research is required, including in vivo studies, to evaluate the toxicity and efficacy of IMBZC in inhibiting the development and metastasis of CRC tumors, understanding the molecular mechanisms behind its anticancer properties, assessing its potential synergistic effects with other chemotherapy drugs or targeted therapies, optimizing its physical and chemical properties, improving its delivery strategies to enhance its effectiveness, and conducting thorough preclinical safety evaluations. If the results are validated, IMBZC could be developed as a novel therapeutic agent or chemosensitizer to improve CRC treatment outcomes.

## Figures and Tables

**Table 1:** Sequences of primer pairs monitoring target gene expression levels using RT-qPCR.

Target Gene	Primer Sequence 5′→3′
*GAPDH*	GTCTCCTCTGACTTCAACAGCG (forward)
	ACCACCCTGTTGCTGTAGCCAA (reverse)
*BAX*	CCCGAGAGGTCTTTTTCCGAG (forward)
	CCAGCCCATGATGGTTCTGAT (reverse)
*BCL2*	TCCGCATCAGGAAGGCTAGA (forward)
	AGGACCAGGCCTCCAAGCT (reverse)
*BCL-xL*	AGTTCCCTTGGCCTCAGAAT (forward)
	TCCTTTCTGGGGAAGAGGTT (reverse)
*TP53*	CCTCAGCATCTTATCCGAGTGG (forward)
	TGAGGCTCACGTCCATCTCGTC (reverse)

## Data Availability

Derived data supporting the findings of this study are available from the corresponding authors (LM and MA) on request.

## Abbreviations

ATCC: American Type Culture Collection; Bax, B-cell lymphoma 2-associated X; Bcl-2, B-cell lymphoma 2; Bcl-xL, B-cell lymphoma—extra large; cDNA, complementary DNA; CRC, colorectal cancer; DMEM, Dulbecco’s modified Eagle’s medium; DMF, dimethylformamide; FBS, fetal bovine serum; GAPDH, glyceraldehyde-3-phosphate dehydrogenase; 5-FU, 5-fluorouracil; HEK, human embryonic kidney; HRP, horseradish peroxidase; IC_50_, half-maximal inhibitory concentration; IMBZC, 1-(Isobutyl)-3-(4-methylbenzimidazolium chloride); IR, infrared; IRI, irinotecan; mCRC, metastatic CRC; MTT, 3-(4,5-dimethylthiazol-2-yl)-2,5-diphenyl tetrazolium bromide; NHC, N-heterocyclic carbene; NMR, nuclear magnetic resonance; OXA, oxaliplatin; PBS, phosphate-buffered saline; PVDF, polyvinylidene fluoride; RIPA, radio-immunoprecipitation assay; RT-qPCR, reverse transcription–quantitative polymerase chain reaction; SD, standard deviation; SDS-PAGE, sodium dodecyl sulfate–polyacrylamide gel electrophoresis; TP, tumor suppressor.
